# Omicron BA.2 Lineage, the “Stealth” Variant: Is It Truly a Silent Epidemic? A Literature Review

**DOI:** 10.3390/ijms23137315

**Published:** 2022-06-30

**Authors:** Giorgio Tiecco, Samuele Storti, Stefania Arsuffi, Melania Degli Antoni, Emanuele Focà, Francesco Castelli, Eugenia Quiros-Roldan

**Affiliations:** Unit of Infectious and Tropical Diseases, Department of Clinical and Experimental Sciences, ASST Spedali Civili di Brescia, University of Brescia, 25123 Brescia, Italy; g.tiecco@unibs.it (G.T.); s.storti@unibs.it (S.S.); s.arsuffi@unibs.it (S.A.); m.degliantoni@unibs.it (M.D.A.); emanuele.foca@unibs.it (E.F.); francesco.castelli@unibs.it (F.C.)

**Keywords:** Omicron, BA.2, lineage, stealth variant, recombinant variant, XE

## Abstract

The epidemic curve of severe acute respiratory syndrome coronavirus 2 (SARS-CoV-2) is silently rising again. Worldwide, the dominant SARS-CoV-2 variant of concern (VOC) is Omicron, and its virological characteristics, such as transmissibility, pathogenicity, and resistance to both vaccine- and infection-induced immunity as well as antiviral drugs, are an urgent public health concern. The Omicron variant has five major sub-lineages; as of February 2022, the BA.2 lineage has been detected in several European and Asian countries, becoming the predominant variant and the real antagonist of the ongoing surge. Hence, although global attention is currently focused on dramatic, historically significant events and the multi-country monkeypox outbreak, this new epidemic is unlikely to fade away in silence. Many aspects of this lineage are still unclear and controversial, but its apparent replication advantage and higher transmissibility, as well as its ability to escape neutralizing antibodies induced by vaccination and previous infection, are rising global concerns. Herein, we review the latest publications and the most recent available literature on the BA.2 lineage of the Omicron variant.

## 1. Introduction

Currently, global attention is focused on the dramatic, historically significant events happening in Ukraine and on the multi-country monkeypox outbreak. However, the epidemic curve of severe acute respiratory syndrome coronavirus 2 (SARS-CoV-2) is silently rising again. Worldwide, the dominant SARS-CoV-2 variant of concern (VOC) is Omicron (phylogenetic assignment of named global outbreak (Pango) lineage designation B.1.1.529), and its virological characteristics, such as transmissibility, pathogenicity, and resistance to both vaccine- and infection-induced immunity as well as antiviral drugs, are an urgent public health concern [1,2]. Omicron (B.1.1.529) was first described in November 2021 during the fourth wave of the SARS-CoV-2 pandemic in South Africa. The initial Omicron variant has further evolved, and the Omicron variant currently includes five major sub-lineages. As of February 2022, the BA.2 lineage has been detected in several European and Asian countries, becoming the predominant variant and the real antagonist of the ongoing surge [1,3,4]. Many aspects of this lineage are still unclear and controversial, but its apparent replication advantage and higher transmissibility, as well as its ability to escape neutralizing antibodies induced by vaccination and previous infection, are rising concerns globally [3]. Herein, we review the latest publications and the most recent available literature on the BA.2 lineage of the Omicron variant.

## 2. Virology and Mutational Profile

**Figure 1:**
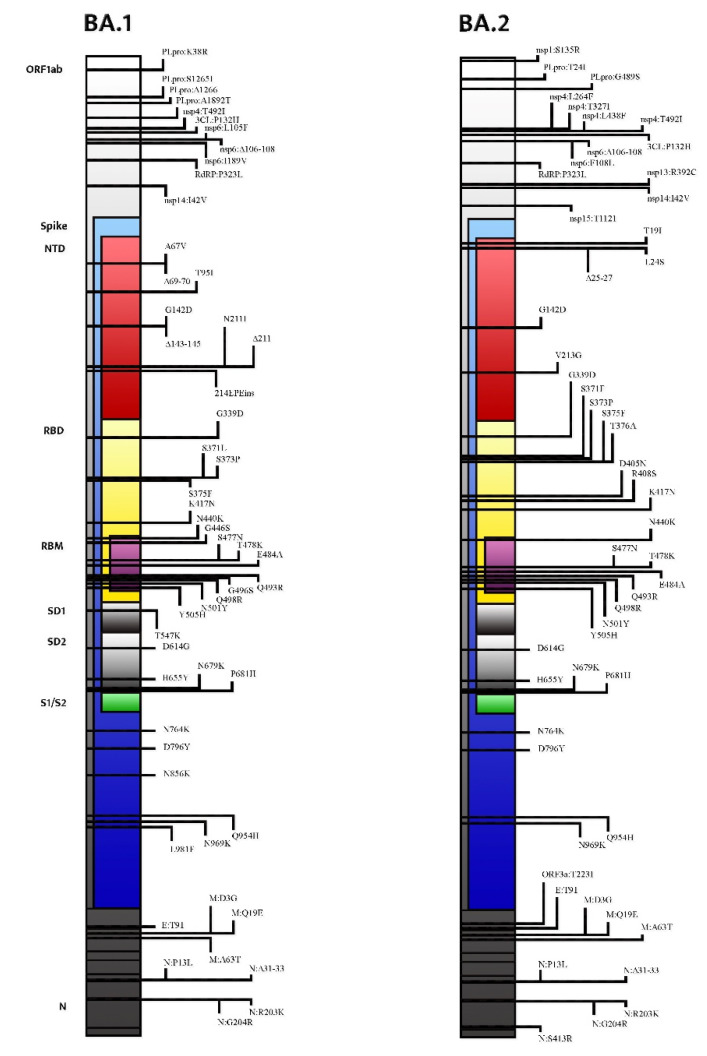
Mutational profile and main differences between the Omicron BA.1 lineage and the Omicron BA.2 lineage.

The Omicron variant lineage consists of several sub-lineages: BA.1 (B.1.1.529 and BA.1.1), BA.2, BA.3, BA.4, and BA.5. Whole-genome sequencing (WGS) analysis identified a total of 50 mutations within the S-protein in the BA.2 variant, compared to 48 mutations present in Omicron BA.1. Additionally, 17 mutations of BA.1 were not present in BA.2 [5]. The differences between the BA.1 and BA.2 lineages and other VOCs are consistent: 28 mutations and 50 amino acids make them approximately twice as different compared to the other four VOCs (Alpha, Beta, Gamma, and Delta) that diverge from the original wild-type SARS-CoV-2 strain [4,5]. Moreover, 17 amino acids, 3 deletions, and 1 insertion (many of them in the N-terminal domain (NTD) and in the receptor-binding domain (RBD)) distinguish BA.1 and BA.2 [6]. Despite growing evidence of the unique aspects of the BA.2 lineage, the WHO stated that BA.2 should be classified as a lineage rather than a VOC (Figure 1) [6]. As Maxmen A. stated, “*all subvariants are variants*”, and researchers use the former term when they want to imply that a lineage belongs to a larger grouping [7]. Currently, the Omicron BA.2 lineage itself accounts for up to 12 branches (as recent analyses are focused on the BA.2.12.1 subvariant) descending from an original Omicron BA.2 ancestor [7].

Phylodynamic analysis states that BA.1 emerged first, followed by BA.2, BA.3, BA.4, and BA.5 [4]. Moreover, the earlier strains of BA.2, BA.3, and BA.1.1 were isolated from Gauteng Province, South Africa [4]. Therefore, it can be deduced that the remarkable diversification of Omicron occurred in Gauteng. Although BA.1 spread worldwide earlier, soon after, the lineage frequency of BA.2 increased; since January 2022, its lineage frequency has exceeded that of BA.1 in several countries [4].

The main characteristic of this variant has several diagnostic reverberations: BA.2, compared to the Delta variant, does not contain the spike gene deletion at positions 69–70, and PCR assays use this S-gene target positivity (SGTP) as a proxy to specifically identify this lineage [8]. However, recently, an increasing number of BA.2 sequences carrying the deletion at 69 and 70 have been identified, suggesting that WGS may be necessary to identify this lineage with certainty [8].

Focusing on the S gene, BA.2 shares 21 mutations with BA.1, but it also has 8 unique mutations prevalently clustered in the N-terminal domain (NTD) and in the receptor-binding domain (RBD) [3]. Compared with BA.1, S371L, G446S, and G496S are deleted in BA.2, whereas S371F, T376A, D405N, and R408S are added [5,9]. The interactions between these unique variations may be responsible for the peculiarities of the BA-2 lineage (Table 1).

As a matter of fact, at the NTD, T19I abrogates a glycosylation site at A27S, significantly changing the antigenic shape of the virus [10,11]. Furthermore, the deletion in the amino acid positions 24–26 and four substitutions in the RBD (S371F, T376A, D405N, and R408S), located in the strategical antigenic site, raise a concern about the possible ineffectiveness of the currently available neutralizing monoclonal antibodies [12,13]. RBD-ACE2 docking under revision studies suggests that all Omicron variants (BA.1, BA.1.1, BA.2, and BA.3) have a greater affinity for the ACE2 receptor because several mutations increase the number of salt bridges and hydrogen bonds between the receptor-binding domain (RBD) and its electronegative receptor (ACE2) [14]. Consequently, antigenic cartography analysis performed to quantify and visualize the antigenic differences between SARS-CoV-2 VOCs have revealed that the BA.2 lineage is qualitatively unique [15]. Considering the Omicron sub-lineage BA.3, it contains 15 mutation sites that are highly consistent with BA.1, while the S371F and D405N mutations take the place of the S371L and G496S mutations in BA.1, respectively [9]. 

Meanwhile, two new members of the Omicron family have been described and named: BA.4 and BA.5. Both variants carry the L452R, F486V, and R493Q mutations, which are involved in the interaction between the RBD and the host cells’ ACE2 receptor [1,16].

## 3. Epidemiology

After a consistent decrease in the epidemic curve, the number of new weekly cases is rising again [1]. Since the first report of the Omicron variant in November 2021, this VOC rapidly displaced the Delta variant, becoming more and more widespread [1]. Among the major Omicron descendent lineages, the BA.2 lineage was detected in Denmark on 5 December 2021 (less than 2 weeks after the first identification of the Omicron variant) [12]. Soon after, in March 2022, the WHO stated that during the seventh week of 2022, BA.2 spread faster than BA.1, becoming the dominant lineage [1]. Beyond what happened in Denmark, this trend was also confirmed in several other countries, including India, the Philippines, and South Africa, where the BA.2 lineage has spread rapidly [17].

At the time of writing, the recent resurgence appeared to be slowing down, with an overall COVID-19 case notification rate for the EU/EEA of 350/100,000; this rate has been decreasing, along with the death rate and ICU admissions. The estimated distribution of Omicron sub-lineages was 83.5% (37.3–97.0% from 11 countries) for BA.2, 8.1% (0.2–61.6% from 10 countries) for BA.5, 2.7% (0.5–8.9% from 9 countries) for BA.4, 12.2% (2.0–16.3% from 4 countries) for BA.2 + L452X, 0.3% (0.0–2.9%, 70 detections from 9 countries) for BA.1, and 0.0% (0.0–0.0%, 2 detections from 1 country) for BA.3 [16]. Similar proportions were reported by the WHO, whose epidemiological update described BA.2 and its descendent lineages as accounting for 44% and 19%, respectively. BA.2.12.1 reached a prevalence of 28%, while BA.4 had a prevalence of 2% and B5 of 4%. BA.1.1, BA.1, and BA.3 account for <0.1% [1]. However, these trends should be carefully interpreted with an eye to surveillance limitations: several countries are progressively changing their testing strategies and sequencing capacities, and their sampling strategies are often different [1].

Exponential models have estimated a daily growth advantage for BA.2 of 0.11 compared to all other Omicron sub-lineages in the U.K.; as of 22 March 2022, according to the U.K. Health Security Agency, the proportion of BA.2 in all recorded infections across the country was 94.7% [18]. Meanwhile, a cross-sectional population-based survey also reported a rapid replacement of Omicron BA.1 and its sub-lineages by BA.2 in the U.K. [19]. It should also be considered that reinfection with BA.2 following infection with BA.1 has been documented: some articles currently under revision sustain that Omicron variants escape neutralizing antibodies elicited by vaccines or previous SARS-CoV-2 infection [20,21].

Concurrently, BA.4 and BA.5 were first detected in South Africa in January and February 2022, respectively, and since then, they have become a major local concern. Additionally, there is an increasing trend in the variant proportions for BA.5 observed in Portugal, accompanied by an increase in COVID-19 case numbers and the test positivity rate. The Portuguese National Institute of Health estimated that BA.5 already accounted for ~37% of positive cases as of 8 May 2022 [16].

## 4. Pathogenesis

The rapid emergence of the BA.2 lineage is associated with its replication advantage, its capacity for immune evasion (compromised serum-neutralizing activity and reduced vaccine effectiveness), and its high transmissibility.

### 4.1. Replication Advantage

The recent increment in the number of BA.2 lineage cases in many countries around the world has suggested that BA.2 has a selective growth advantage over other circulating variants [3,17]. In addition to the previously mentioned U.K. Health Security Agency data, a Bayesian model was constructed to quantify the growth advantage of BA.2, revealing an effective reproduction number 1.40-fold higher than that of BA.1 (95% confidence interval) [4]. This may also be linked to the higher environmental stability shown by the Omicron variants both on plastic and skin surfaces, with approximately two-fold longer survival times when compared to the original Wuhan strain [22]. Although this high replication rate has been confirmed by several organizations around the globe, the growth rates may be overestimated, especially early during the emergence of a new variant [8].

### 4.2. Immune Escape

The ability of BA.2 to evade neutralizing antibodies induced by vaccination or infection is unclear [3]. In New York, both sera from people vaccinated with three doses and sera from patients infected during the Omicron surge produced neutralizing antibodies that were just slightly better at fending off infection by viruses belonging to the BA.1 lineage than BA.2’s [3]. Moreover, in animal models, hamsters and mice infected with BA.1 produced antibodies that were less effective against BA.2, suggesting the in vitro possibility of reinfection [4]. The small difference in overall potency against the two variants means that an ability to evade immunity is unlikely to explain BA.2’s ascent worldwide [17]. Similarly, a recent analysis using pseudo-viruses compared BA.1, BA.2, and BA.3 for sensitivity to neutralization by vaccination- and infection-induced antibodies. In particular, considering sera from convalescent patients infected during the first (February to May 2020) and second (December 2020 to February 2021) waves, the neutralizations of BA.1 and BA.3 were at least 32 times lower (BA.1 *p* = 0.0020; BA.3 *p* = 0.0020) in comparison with the neutralization of B.1 (which was quite similar to the original wild-type strain) [23]. This means that the neutralization of BA.2 was less pronounced than that measured for the other Omicron subvariants (9.2 times less than B.1; *p* = 0.0020). Moreover, BA.1 infection elicited similar levels of cross-neutralization against BA.2 and BA.3, although at a decreased efficiency that was 4.2- to 4.4-fold lower than that against BA.1 [24]. Similarly, double-vaccination-induced neutralization showed a 17-fold reduction when comparing BA.1 or BA.3 to B.1 (BA.1 *p* = 0.0020; BA.3 *p* = 0.0020), whereas the neutralization of BA.2 was just 9-fold reduced (*p* = 0.0020) [23]. A study currently under revision has proposed a mechanism to explain the BA.2 lineage’s broad immune escape: the S371L/F mutation in the RBD seems to induce dynamic conformational changes of the spike trimer, reducing antibody neutralization without detrimental effect on viral fitness [25]. However, according to another analysis, the immune escape capacity of BA.2 seems to be less effective in comparison to that of BA.1 [26]. Other viral or host factors are perhaps involved in driving the rapid surge of this new lineage.

Real-world data from an experiment in Israel showed that unvaccinated, double-vaccinated, and boosted individuals were found to be more susceptible to BA.2 infection than to BA.1 infection [17]. Although rare, Omicron BA.2 reinfections in vivo may occur, especially shortly after BA.1 infections [20]. In addition, in antigen-naïve individuals, the immunologic response following infection with the BA.2 lineage is lower than that following BA.1 infection [27]. This may have crucial global implications as a lower neutralization response may contribute to the prolonged circulation of the virus in the population [27].

Moreover, the ECDC has raised awareness regarding BA.4 and BA.5 emergence due to the limited availability of data from in vitro studies evaluating sera from unvaccinated individuals who have experienced a prior BA.1 infection, which indicates that both BA.4 and BA.5 are capable of escaping the immune protection induced by infection with BA.1 [28]

### 4.3. Increased Transmissibility

It is known that the Omicron variant has a higher affinity for the ACE2 receptor and, consequentially, the potential for increased transmission [2]. The hACE2 receptor bound to the Omicron BA.2 spike trimer with a dissociation constant approximately 11-fold higher than that for the WT spike trimer and nearly 2-fold higher than that for the BA.1 spike trimer [29]. Among the Omicron sub-lineages, BA.2 and BA.3 have higher transmission potentials compared to BA.1: BA.2 has a docking energy of −974, which is higher than that of BA1.1 (−946.8) and BA.1 (−943.4) but lower than that of BA.3 (−999.3) [12]. However, according to a preprint analysis carried out in Denmark, the difference in terms of the transmission rate appears to be less than that between Delta and Omicron [30]; nevertheless, an enhanced attractivity towards the strongly electronegatively charged ACE2 receptor protein can still be predicted [31]. An algebraic topology-based model was used to evaluate the infectivity of the Omicron sub-lineage. It was estimated that BA.2 was approximately 20, 4.2, and 1.5 times as infectious as the ancestral SARS-CoV-2 wild-type strain, the Delta variant, and BA.1, respectively [32].

The RT-qPCR cycle threshold (Ct) value of a SARS-CoV-2 infection represents the inverse of viral load, and it can be used as a proxy for SARS-CoV-2 infectiousness. BA.2 was associated with 3.53 fewer cycles (95% CI: 3.46–3.60) when compared to BA.1, signifying higher infectiousness [33].

An interesting experiment that highlighted the exceptionally high transmissibility of the BA.2 lineage occurred in a single housing estate in Hong Kong. This outbreak, which was fortunately promptly controlled with the lockdown of three buildings, had a very short doubling time of 1.28 days (95% confidence interval: 0.560–1.935), and the phylogenetic analysis showed that these sequences clustered together [34].

To sum up, it is logical to wonder whether increased transmissibility or immune escape best explain the Omicron BA.2 surge. No conclusive studies are currently available. However, in vitro entry assay studies state that Omicron variant infection is not enhanced by TMPRSS2, and no particular difference has been seen between Delta and Omicron entry that might explain the overtake of Delta by Omicron [35]. This result, together with the Danish real-world data [30], tends to indicate that the increased transmissibility of Omicron over Delta may be more a consequence of increased immune evasion.

## 5. Clinical Manifestations and Diagnosis

Data on BA.2 lineage severity are controversial, and greater challenges may be posed in places with lower vaccination coverage [17]. A study in Japan showed that, in animal models without any immunity to SARS-CoV-2, BA.2 might have caused more severe disease [36]. In particular, studies under revision that used animal models with B.1.351-infected mice resulted in significant changes in bronchoconstriction; however, the BA.2-infected mice exhibited only a localized, limited, and attenuated inflammatory response, although significantly higher levels of pro-inflammatory cytokines (IL-1β, IFN-γ, MIP-1β) were recorded compared to those of mice infected with BA.1 [37]. Moreover, this study showed that BA.1 became dominant in the upper airways, whereas both the BA.1 and BA.2 lineages were found in the bronchiolar and alveolar epithelium [37].

Real-world data (where immunity from vaccination and natural infection is high) reported no significant difference in severity between BA.2 and BA.1 with regard to age, sex, SARS-CoV-2 reinfection, hospitalization, or mortality, suggesting that BA.2 leads to an equally mild course of disease [12]. However, other studies suggest that people over the age of 80 seem to be more susceptible to severe infections when infected by the BA.2 lineage [38]. Interestingly, BA.2 infection has a shorter average time from symptom onset, as demonstrated by contact tracing data: the mean onset interval is around half a day shorter for BA.2 than BA.1 (3.27 days for BA.2, 3.72 days for BA.1, and 4.09 days for Delta) [8]. It is known that, in contrast to previous variants, the Omicron variant affects all age groups, including the youngest [39,40]. The “seven-day incidence indicator” that describes the number of infections within 7 days per 100,000 inhabitants is currently the highest among the 5–14-year-old age group [39]. Similarly, according to the CDC, the number of young children hospitalized in the U.S.A. due to COVID-19 is rising; however, it is reasonable to say that many children who tested positive for SARS-CoV-2 are currently coming to hospitals with other respiratory illnesses [8].

Lastly, when comparing the hospitalization odds between Delta and Omicron independently of vaccination status, the hospitalization frequency among Delta cases seemed nearly three-fold higher (8.3%) than for Omicron (3.0% for both lineages BA.1 and BA.2), suggesting that Omicron inherently causes less severe disease [41].

Regarding diagnosis, it is known that the BA.2 lineage was nicknamed the “stealth variant” because of the erratic presence of the S-gene target failure (SGTF) on diagnostic assays [8]. As mentioned before, the spike gene deletion at positions 69–70, which is characteristic of Omicron BA.1, is frequently absent in BA.2 strains [8]. This means that while the Omicron variant BA.1 lineage was easily differentiated from the Delta variant through PCR assays, the BA.2 lineage was often indistinguishable from Delta in areas where both VOCs were circulating [42]. In other words, a person infected by BA.2 tests positive for SARS-CoV-2 on a PCR test, but genetic sequencing is necessary to flag the case as caused by the BA.2 lineage [8]. Nevertheless, a recent study tested partial ORF1ab gene target failure (pOGTF) on the cobas SARS-CoV-2 assays, demonstrating a 98.6% sensitivity and a 99.9% specificity for the SARS-CoV-2 lineage BA.2.12.1 [43]. Consistent results prove that antigenic tests are a viable alternative for detecting the Omicron variant. However, few data referred to the BA.2 lineage are currently available [44], although several commercially available rapid antigen test kits may detect Omicron BA.2 with discreet analytical sensitivity [45].

## 6. Treatment

### 6.1. Antivirals

The antiviral drugs active against the Omicron variant of SARS-CoV-2 are inhibitors of the RNA-dependent RNA polymerase (RdRp) and of the 3CLpro (3C-like protease) that is the main protease of the virus. Despite several mutations described in the Omicron variant, these molecules seem to maintain activity against this newly emerged VOC [2]. Remdesivir and molnupiravir are both nucleoside analogues that inhibit the RdRp of *Coronavirdae*, while nirmatrelvir and other drugs currently in clinical trials, such as S-217622 (phase 3), are inhibitors of 3CLpro [13,46]. The effects against BA.2 of approved antiviral drugs and those under investigation are still uncertain.

The in vitro susceptibility of BA.2 to remdesivir, molnupiravir, and nirmatrelvir appears to be similar to that of the ancestral strain and other variants of concern [13,47]. Moreover, in animal models, the therapeutic efficacy of these compounds was assessed in hamsters infected with BA.2. There was no decrease in viral titers in the nasal turbinates of the animals treated with molnupiravir or nirmatrelvir, while treatment with S-217622 reduced the number of virus titers in the upper respiratory tract. Notably, all the compounds tested considerably reduced the number of lower respiratory tract virus titers [37].

The few data available suggest that antiviral drugs remain active against BA.2, even though clinical data and real-world experiments are not currently available.

### 6.2. Monoclonal Antibodies

The use of monoclonal antibodies against SARS-CoV-2 has characterized the recent treatment scenario [48]. BA.2 appeared to retain in vitro sensitivity to some NTD-targeting mAbs. Mutations in the N1-loop moderately decreased binding for class II, III, and IV mAbs and, in combination with G142D, BA.2 completely escaped antibody recognition at this site. Thus, BA.1 and BA.2 effectively evaded class III and IV mAbs but showed different binding for class I and II mAbs.

Mutations in both the BA.1- and BA.2-RBDs often resulted in non-additive levels of escape from RBD-targeting mAbs. Multiple mutations were often required to escape binding completely [49]. The in vitro inhibition potency of casirivimab, imdevimab, and S309 with respect to Omicron subvariants BA.1 and BA.2 was severely impaired, in contrast to the outstanding activity of these treatments against the Wuhan strain and the Alpha, Gamma, and Delta variants. In this under-revision analysis, casirivimab did not show any antiviral activity for BA.1 or BA.2. In addition, imdevimab lost its activity against BA.1, but retained a minor level of antiviral activity that allowed it to reduce BA.2 infections. S309’s antiviral activity against BA.1 was more modest than that of other variants, and its activity against BA.2 was even weaker [47]. However, BA.2 may have limited management options. Although the drug’s manufacturer claimed that sotrovimab (GSK4182136 or S309) remained effective against BA.2, recent analysis suggests that the BA.2 lineage is intrinsically resistant to this monoclonal antibody [17,50,51]. As a matter of fact, the Federal Drug Administration (FDA) decided to limit the use of sotrovimab in the treatment of mild-to-moderate COVID-19 [52]. Currently, this monoclonal antibody is specifically authorized in geographic regions where infection is not likely to have been caused by the BA.2 lineage or after its certain exclusion [52]. Similarly, both etesevimab (LY-CoV016) and bamlanivimab (LY-CoV555) showed a markedly reduced neutralizing activity against the BA.2 lineage in live-virus tests [53]. There are high hopes for combination therapies. Despite its ineffectiveness against the Omicron BA.1 lineage, the well-known combination of casirivimab (REGN10933) and imdevimab (REGN10987) may inhibit the BA.2 lineage [53,54]. Likewise, another effective combination against the BA.2 lineage is tixagevimab (AZD8895) and cilgavimab (AZD1061): this combination has shown an effective synergistic effect, as supported by the analysis of spike protein-pseudo-typed lentiviruses [54,55,56]. It is noteworthy that the monoclonal antibody titer required for a 50% reduction in the number of infections has risen [53]. These new findings show that none of the currently approved or authorized monoclonal antibody therapies adequately cover all the sub-lineages of the Omicron variant [50]. Finally, a preprint study identified six mAbs that bound to the spike protein from all human alpha- and beta- coronaviruses. These mAbs target the fusion peptide region, which plays a critical role during coronavirus invasion, has an identical sequence in all SARS-CoV-2 VOCs, and is highly conserved. However, these mAbs have a significantly low in vitro neutralization potency that may be due to their relatively weak binding to the intact spike, which is enhanced only when the S1 cap is removed [57]. Many analyses are currently under revision, and one of them states that LY-CoV1404 (also known as bebtelovimab) displays potent neutralizing activity against numerous variants, including the BA.2 subvariant, as it retains binding activity to the spike proteins despite several underlying RBD mutations (K417N, L452R, E484K, and N501Y) [58].

### 6.3. Vaccines

The different Omicron lineages are antigenically distant from wild-type SARS-CoV-2, and this threatens the efficacy of currently available vaccines [50]. As a matter of fact, polyclonal sera from patients infected by wild-type SARS-CoV-2 showed a substantial loss in neutralizing activity against the BA.2 lineage [50]. Moreover, recent studies confirmed that neutralizing titers were lower against BA.2 compared with ancestral SARS-CoV-2 after one or two doses, as well as boosted vaccination [59,60].

However, vaccine efficacy appears to be similar against BA.2 in comparison to BA.1 and the other Omicron sub-lineages [8]. A case-control study evidenced no reduction in vaccine effectiveness against symptomatic disease or hospitalization with BA.1 and BA.2 after one or two doses of BNT162b2, ChAdOx1-S, or mRNA-1273, and after booster doses of BNT162b2 or mRNA-1273 during a period of co-circulation [61]. Recent data suggest that the mRNA vaccines induce the highest antibody titer against the Omicron variant, but a booster dose is needed to obtain consistent neutralizing antibody titers against either the BA.1 or BA.2 lineages [46,47]. A recently published, matched, test-negative, case-control study estimated the duration of protection of the second and third/booster doses of BNT162b2 and mRNA-1273 against Omicron BA.1 and BA.2. In the first three months after the second dose, effectiveness was high against both the lineages considered, but it declined to ~10% or below thereafter. In contrast, in the first month after the booster dose, effectiveness rebounded to high levels of protection with an efficacy against COVID-19 hospitalization and death of >90% [62].

Particles harboring the S proteins of BA.2 and BA.3 showed lower neutralization than those of B.1 when exposed to vaccinated or recovered patients’ sera. Triple BNT vaccination induced a potent antibody response, and only a modest evasion of neutralization was seen for particles bearing Omicron BA.2 and BA.3 S proteins. Neutralization by the antibodies induced in fully-vaccinated (three vaccine doses) individuals with breakthrough infections during the fourth wave in Germany (October 2021 to January 2022, dominated by the Delta variant) showed no significant differences between BA.1, BA.2, and BA.3 [56]. There is controversial data regarding the use of an Omicron-based vaccine (mRNA-1273.529 or Omicron-BA.1-RBD) in a complete vaccination cycle based on mRNA vaccines. Some analyses conducted in mice suggest that it seems to increase neutralizing titers and protection against BA.1 and BA.2 without less effectively inhibiting historical or other SARS-CoV-2 variants [55,63], whereas other studies suggest that an Omicron boost may not provide greater immunity or protection compared to a boost with the current mRNA-1273 vaccine [21,64]. Lastly, important public health implications were derived from the discovery that sera of patients previously infected with the BA.1 lineage seemed to have robust neutralizing antibody titers against BA.2, suggesting a substantial degree of cross-reactive natural immunity [3]. Considering the cellular response to vaccines, data under revision suggested that the introduction of a serine at position 446 of the Omicron BA.1 spike protein would be sufficient to induce enhanced T cell recognition of the NF9 epitope. This enhanced recognition was diminished against the Omicron BA.2 spike protein due to the absence of the G446S mutation. In contrast, QI9-specific T cells from the same donors produced comparable levels of IFN-γ in response to stimulation with target cells expressing all spike proteins tested [65].

## 7. Conclusions

The evolution of SARS-CoV-2 has always been characterized by the emergence of sets of new mutations impacting the virus’s main features. Therefore, the emergence and spread of the Omicron BA.2 lineage were enabled by its unique abilities: flexibility of transmission, replication advantage, and significant neutralization escape [66]. Moreover, this new surge has again demonstrated that a “one pill fits all” strategy does not exist for SARS-CoV-2 or, more specifically, for the Omicron variant [50]. Measures complementary to vaccination and the treatment options previously mentioned, such as the use of masks, hand hygiene, and keeping environments ventilated, are still essential to delay the emergence of new variants [67].

**Figure 2:**
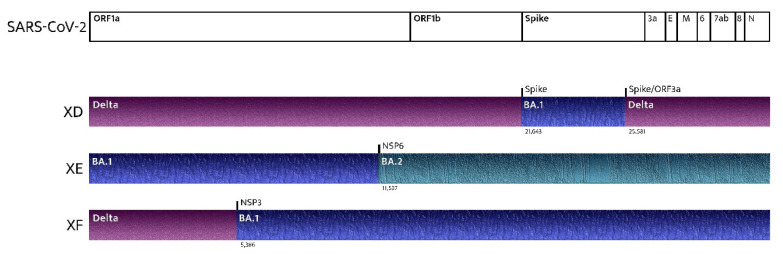
Recombinant SARS-CoV-2 variants: where do they come from?

Frequently, while the dominant strain is declining, other circulating variants start to make room for themselves. In this scenario, multiple variants may infect the same host, generating recombinant variants [68]. The spreading of recombinant SARS-CoV-2 variants is a phenomenon currently occurring, and it is raising concerns in several countries [8,69]. XD and XF (recombinations of the BA.1 and Delta variants) are responsible for several clusters in Europe. However, the most concerning variant is XE (a recombination of the BA.1 and BA.2 lineages of the Omicron Variant) because of its growth rate, which is 9.8% above that of BA.2 (Figure 2) [70,71,72]. The earliest confirmed case of XE is dated 19 January, and it has been detected in the U.K., Thailand, India, Israel, Japan, and Italy. Cases of the new XE strain are increasing quickly and have nearly doubled in the U.K. during the last few weeks, according to the latest data from the U.K. Health Security Agency. About 1125 cases of XE have been identified as of 5 th April [18]. Few data are available on a new recombinant sequence of the SARS-CoV-2 Omicron variant detected in Finland: the recombinant XJ lineage seems to have a 5’-end of BA.1, a recombination breakpoint between ORF1a and ORF1b, and a 3’-end of BA.2 [73].

On 14 April, the WHO Africa announced that research in Botswana and South Africa had detected new sub-lineages of the Omicron variant, namely BA.4 and the BA.5, that had previously been identified outside Africa in Belgium, Denmark, Germany, China, France, Portugal, and the United Kingdom [74]. Scientists are now studying these subvariants to determine whether their effects are serious enough to warrant interventions.

In view of the above statement and ambiguous points, although global attention is currently focused on other dramatic, historically significant events and although political, economic, and social needs exert pressure for a return to the pre-COVID-19 era, this new surge may not be a silent epidemic. The continual evolution of SARS-CoV-2 challenges the available therapies and vaccines. Investments in new drugs and worldwide vaccination programs, with a special focus on developing countries, must be incentivized to reach the goal of eradication.

## 8. Research Strategy and Selection Criteria

References for this review were identified from PubMed, Embase, and Cochrane with the following research terms: “Omicron BA.2”, “Omicron/BA.2”, “B.1.1.529.2”, and “Omicron sub-lineage”. These key words were severally combined with “mutations”, “diagnosis”, and “treatment”. Only papers in English were included. This is a non-systematic review, and the final reference list was generated based on timeline, originality, and relevance to the scope of this review.

## Figures and Tables

**Table 1:** Comparison between the BA.1 and BA.2 mutational profiles in the SARS-CoV-2 spike. Abbreviations used: NTD (N-terminal domain), RBD (receptor-binding domain), RBM (receptor-binding motif), SD1 (subdomain 1), SD2 (subdomain 2), FP (fusion peptide), and HR (heptad repeat).

	Shared among BA.1 and BA.2	Exclusively in BA.1	Exclusively in BA.2
**NTD**	G142D	A67V, ∆69–70, T95I, ∆143–145, N211I, ∆211, 215EPEins	T19I, ∆24–26, A27S, V213G
**RBD**	G339D, S373P, S375F, K417N	S371L	S371F, T376A, D405N, R408S
**RBM**	N440K, S477N, T478K, E484A, Q493R, Q498R, N501Y, Y505H	G446S, G496S	
**SD1**		T547K	
**SD2**	D614G, H655Y, N679K, P681H		
**FP**	N764K, D796Y	N856K	
**HR**	Q954H, N969K	L981F	

## Data Availability

Not applicable.
